# Supplementary material for: A twelve-electron conversion iodine cathode enabled by interhalogen chemistry in aqueous solution
Source: Nat Commun. 2023 Sep 7;14:5508. doi: 10.1038/s41467-023-41071-6 (PMC10484974; doi:10.1038/s41467-023-41071-6)
Supplement: Supplementary file 1 — Supplementary Information [file 41467_2023_41071_MOESM1_ESM.pdf]

Supplementary Information

A twelve-electron conversion iodine cathode enabled by interhalogen chemistry in aqueous solution

Wenjiao Ma[1], Tingting Liu[1], Chen Xu[1], Chengjun Lei[1], Pengjie Jiang[1], Xin He[1] and Xiao Liang[1*]

1 State Key Laboratory of Chem/Bio-Sensing and Chemometrics, Advanced Catalytic Engineering Research Center of the Ministry of Education, College of Chemistry and Chemical Engineering, Hunan University, Changsha 410082, China

* Corresponding author: xliang@hnu.edu.cn

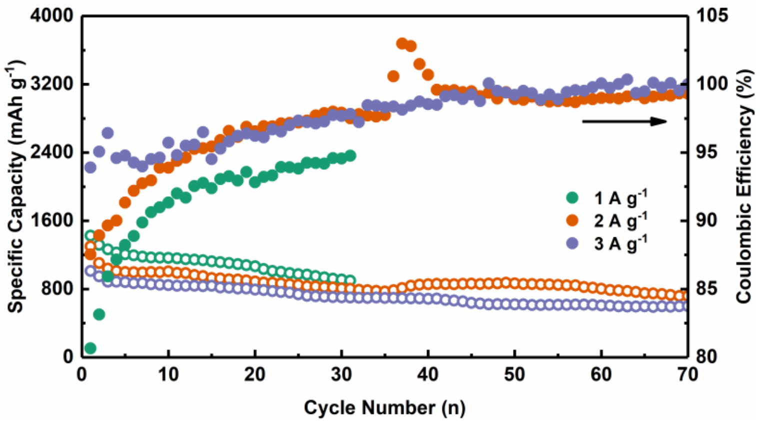

Supplementary Fig. 1 Cycling performance of I_2/HAC electrode in 0.1 M H_2SO_4 + 0.1 M KBr electrolyte at 1, 2 and 3 A g^{-1}.

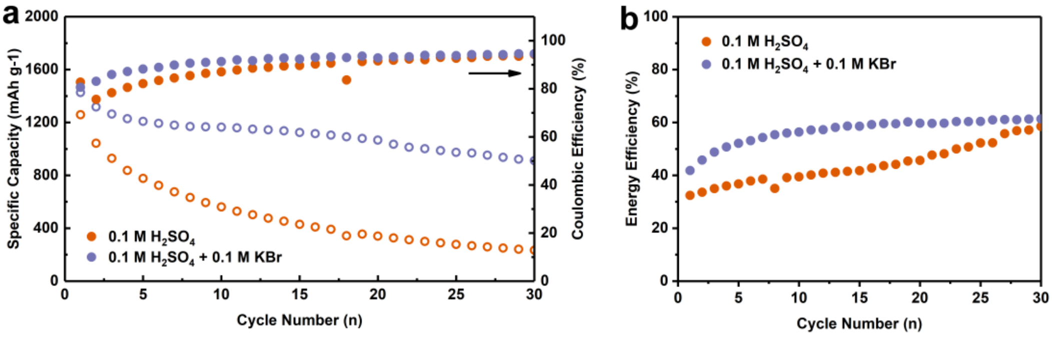

Supplementary Fig. 2 The effect of Br^-/Br_2 redox mediator on the I^-/IO_3^- redox couple. **(a)** Cycling performance and **(b)** energy efficiency of I_2/HAC electrode at a current density of 1 A g^{-1}. The electrolyte was 0.1 M H_2SO_4 or 0.1 M H_2SO_4 + 0.1 M KBr.

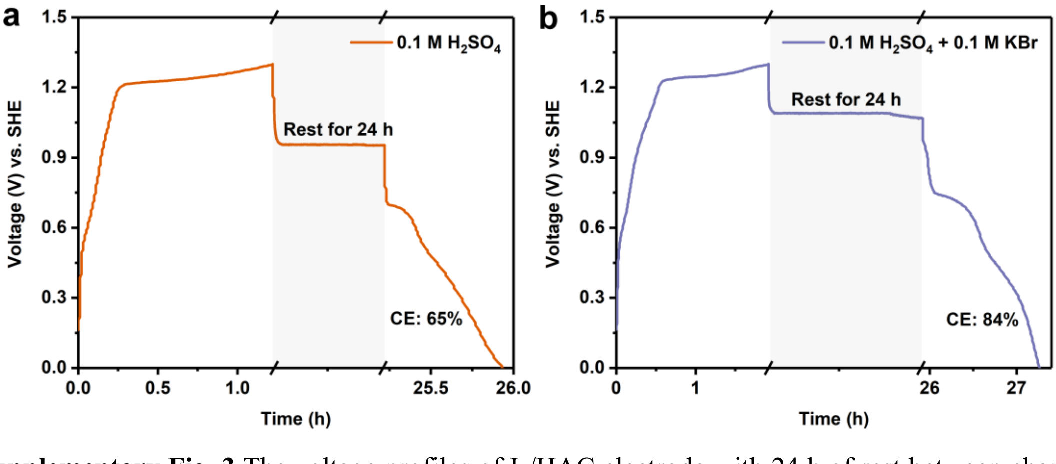

Supplementary Fig. 3 The voltage profiles of I_2/HAC electrode with 24 h of rest between charge and discharge process at 1 A g^{-1} in **(a)** 0.1 M H_2SO_4 and **(b)** 0.1 M H_2SO_4 + 0.1 M KBr electrolyte.

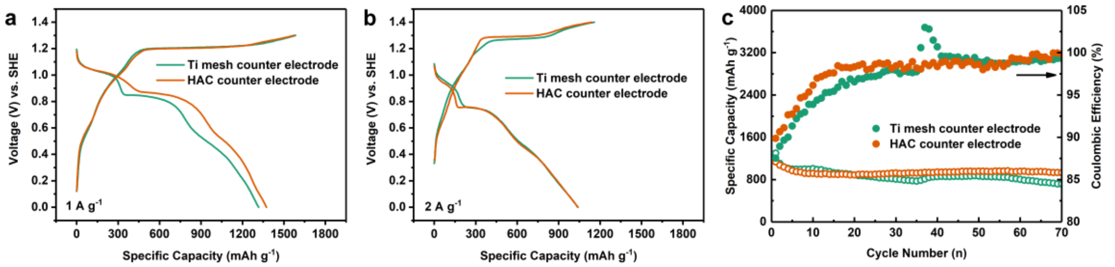

Supplementary Fig. 4 Voltage profiles of I_2/HAC electrode at **(a)** 1 A g^{-1} and **(b)** 2 A g^{-1}. **(c)** Cycling performance of I_2/HAC electrode at 2 A g^{-1}. The electrolyte was 0.1 M H_2SO_4 + 0.1 M KBr.

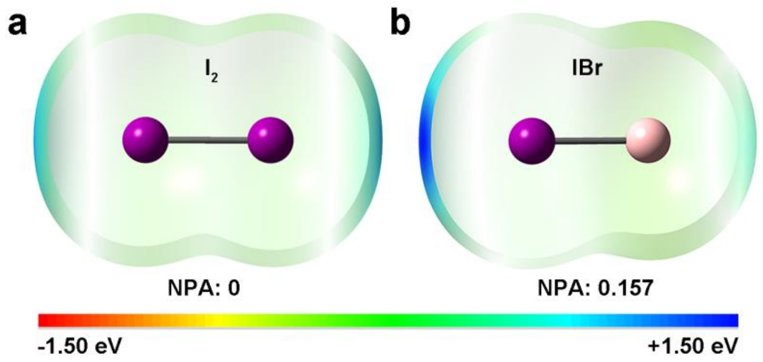

Supplementary Fig. 5 The ESP of **(a)** I_2 and **(b)** IBr molecules (the purple ball represents iodine atom, and the pink ball represents bromine atom). The NPA represents the natural population analysis of the iodine atom.

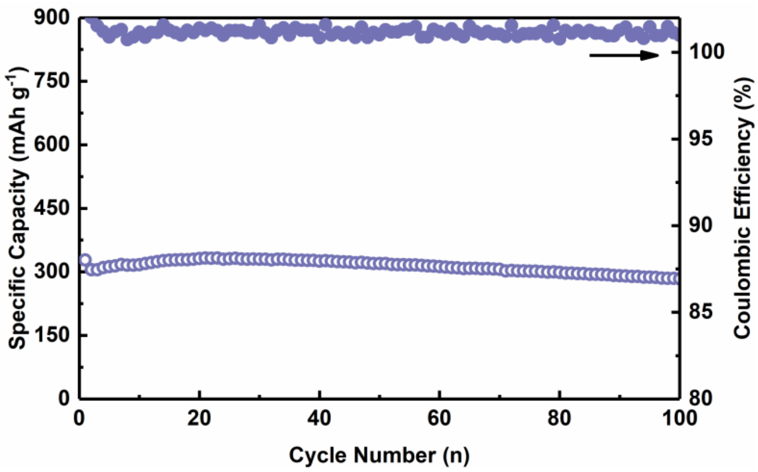

Supplementary Fig. 6 Cycling performance of the I_2/HAC electrode in 0.1 M H_2SO_4 + 0.1 M KBr electrolyte at 1 A g^{-1}. The cell was tested at 0 ~ 1 V vs. SHE.

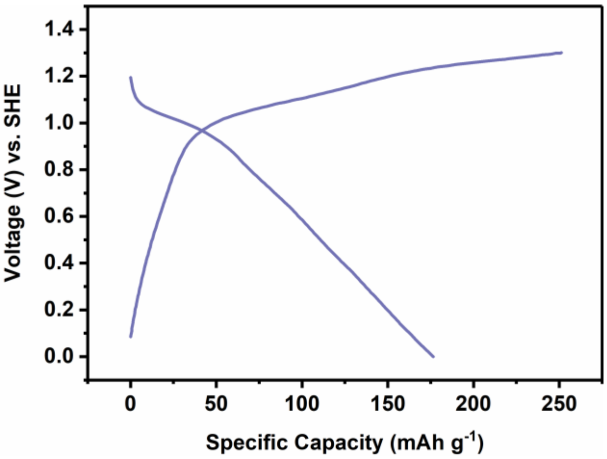

Supplementary Fig. 7 Voltage profile of the HAC electrode in 0.1 M H_2SO_4 + 0.1 M KBr electrolyte. The specific capacity is calculated based on the average mass loading of iodine (1.5 mg cm^{-2}).

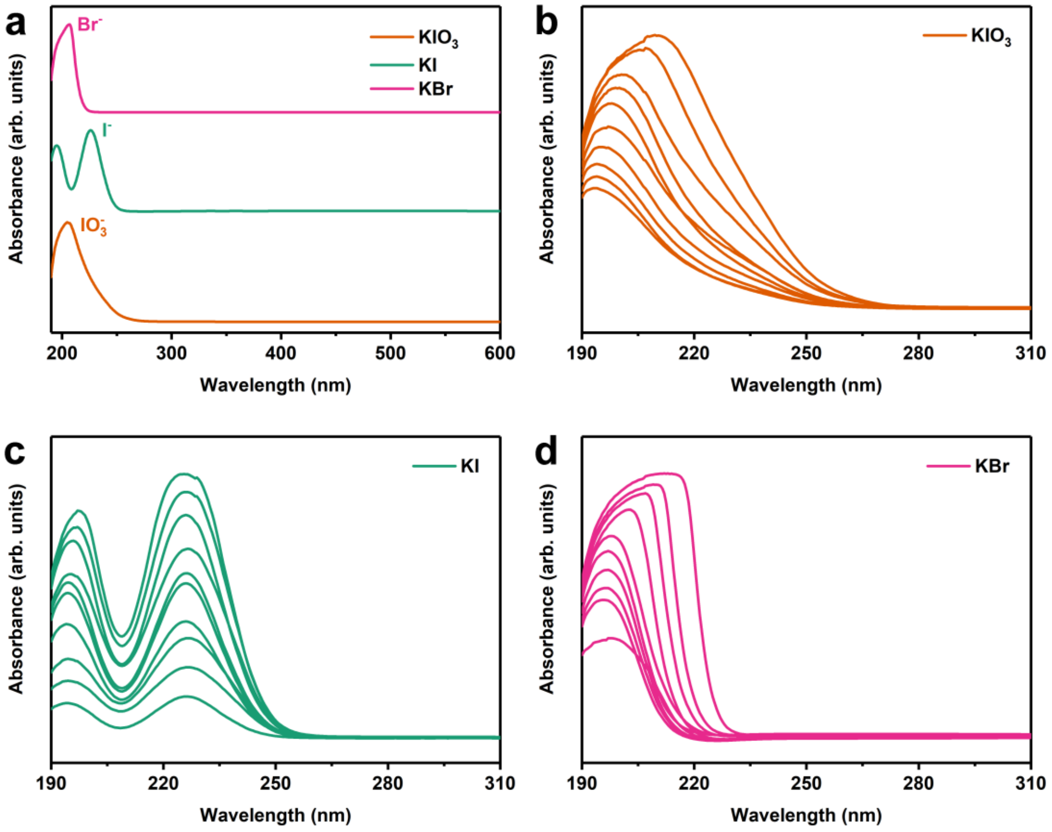

Supplementary Fig. 8 (a) UV-vis spectra of the IO_3^-, I^- and Br^-. UV-vis spectra of the **(b)** KIO_3, **(c)** KI and **(d)** KBr standard solution with different concentrations. The peak position of IO_3^- and Br^- have obvious variation as the concentration changes, whereas the peak position of I^- does not vary with the concentration.

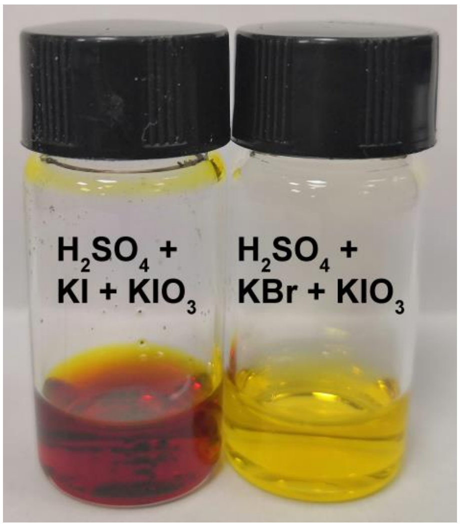

Supplementary Fig. 9 The color of the solution of H_2SO_4 + KI + KIO_3 and H_2SO_4 + KBr + KIO_3. All of the H_2SO_4 + KI, H_2SO_4 + KBr and KIO_3 solutions are colorless before contact. It turned brown or yellow because of the chemical reaction between I^-/Br^- and IO_3^- in the presence of acid.

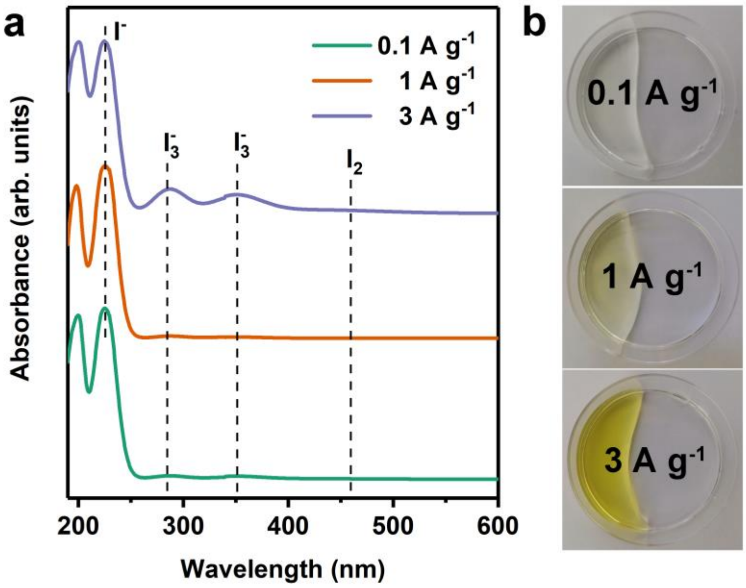

Supplementary Fig. 10 The formation of IO_3^- at different current densities was demonstrated by the chromogenic reaction with iodide. **(a)** UV-vis spectra of the E-extracted solution at different current densities after the color titration in 0.1 M H_2SO_4 + 0.1 M KBr electrolyte. **(b)** The corresponding color titration of the E-extracted solution in 0.1 M H_2SO_4 + 0.1 M KBr electrolyte. The E-extracted solution was obtained at the end of first discharge plateau.

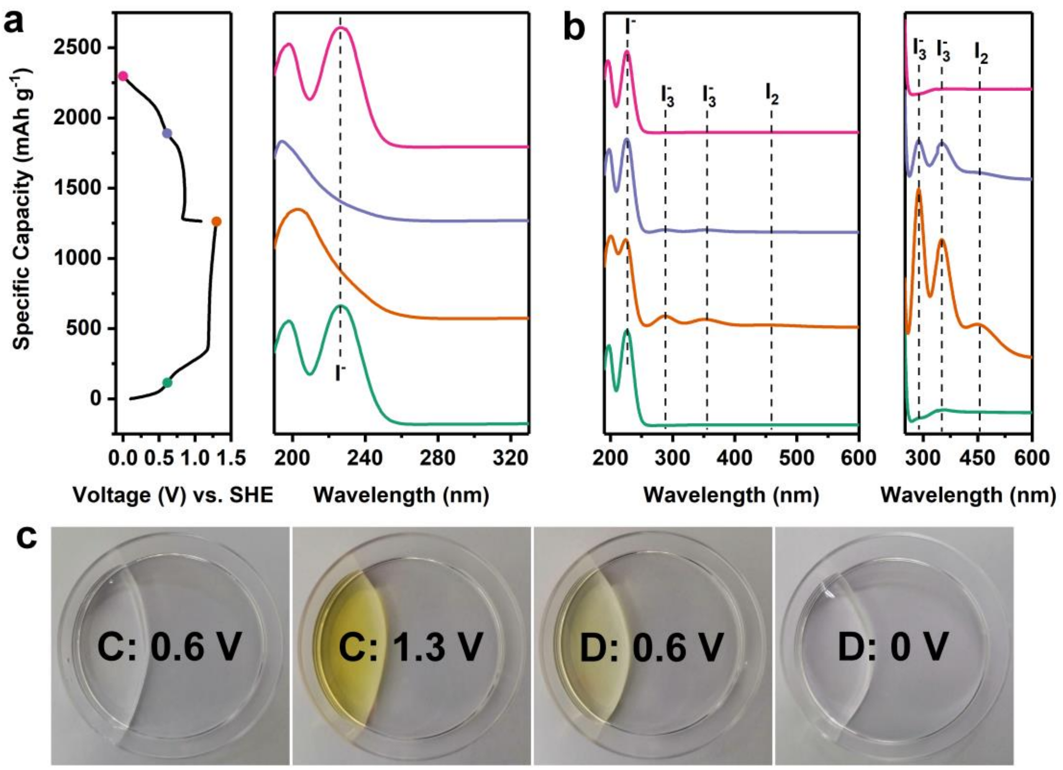

Supplementary Fig. 11 UV-vis spectra of the E-extracted solution **(a)** during the charge-discharge process and **(b)** after the color titration in 0.1 M H_2SO_4 electrolyte. **(c)** The color titration of the E-extracted solution been tested in 0.1 M H_2SO_4 electrolyte (C and D represents the charge and discharge process, respectively).

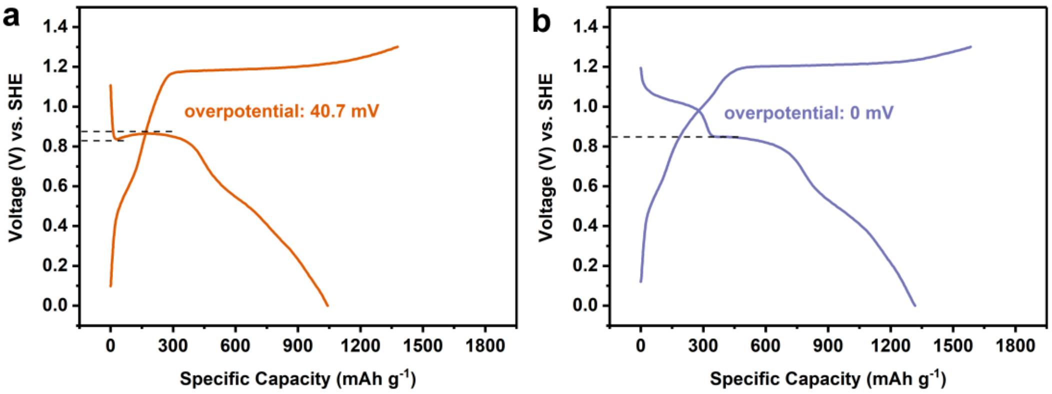

Supplementary Fig. 12 The overpotential of I_2 deposition in **(a)** 0.1 M H_2SO_4 and **(b)** 0.1 M H_2SO_4 + 0.1 M KBr electrolyte at 1 A g^{-1}.

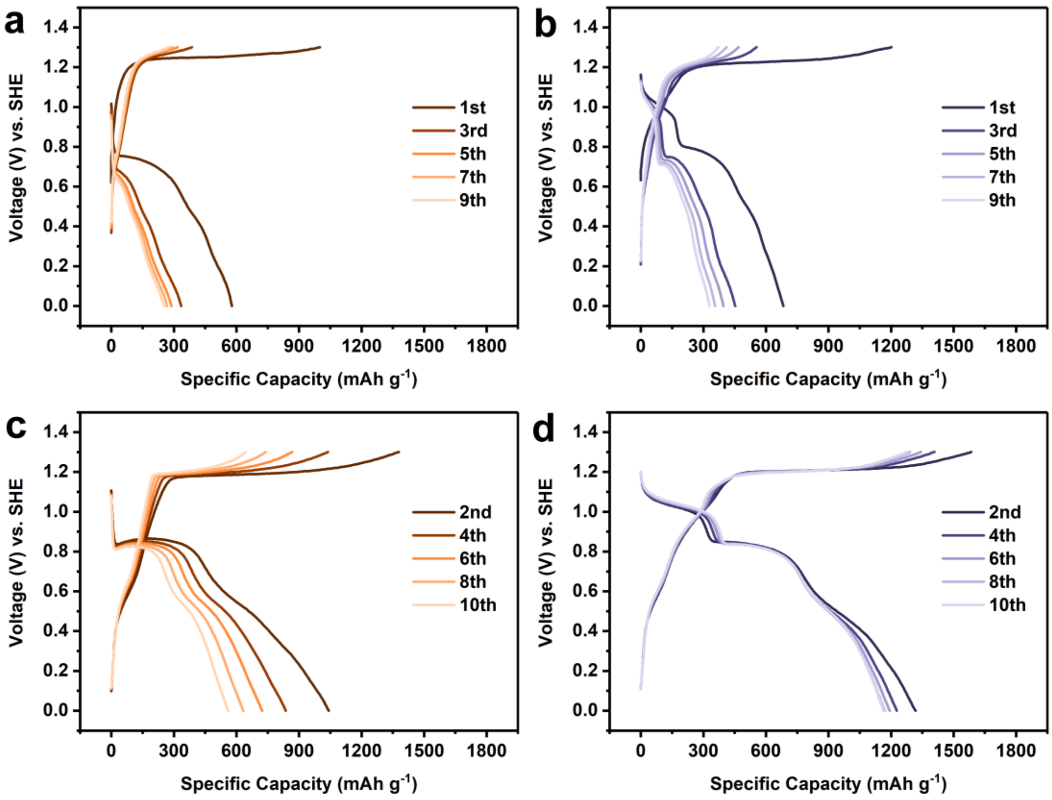

Supplementary Fig. 13 Voltage profiles of I_2/HAC electrode in **(a)** 0.1 M K_2SO_4, **(b)** 0.1 M K_2SO_4 + 0.1 M KBr, **(c)** 0.1 M H_2SO_4 and **(d)** 0.1 M H_2SO_4 + 0.1 M KBr electrolyte at 1 A g^{-1}.

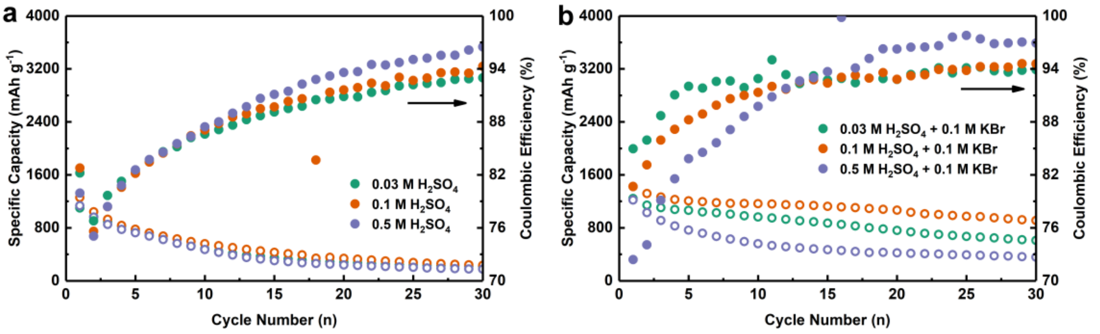

Supplementary Fig. 14 Cycling performance of I_2/HAC electrode in **(a)** H_2SO_4 electrolyte with various concentrations and **(b)** 0.1 M KBr supported electrolyte with different H_2SO_4 concentrations. The current density was 1 A g^{-1}.

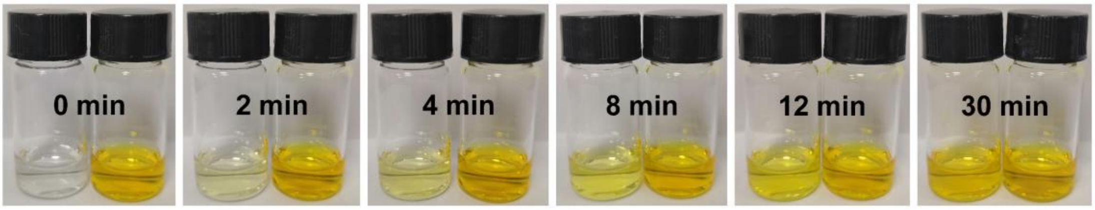

Supplementary Fig. 15 The influence of reaction speed of iodate with different KBr concentrations. The color of solution was changed from colorless to yellowish with time when 0.1 M KBr (on the left) and 0.5 M KBr (on the right) drop into 0.1 M H_2SO_4 + 0.1 M KIO_3 solution, respectively. The color of solution with low Br^- concentration changes slowly because of the slow chemical reaction between Br^- and IO_3^-.

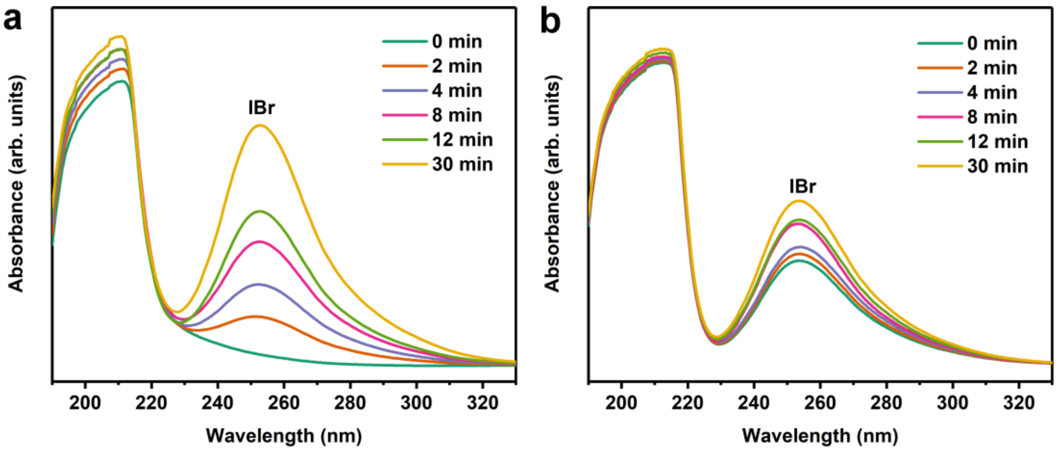

Supplementary Fig. 16 Time dependent UV-vis spectra of the Br^- + IO_3^- reaction with different KBr concentrations. The peak intensity of IBr increases gradually with time when **(a)** 0.1 M and **(b)** 0.5 M KBr drop into 0.1 M H_2SO_4 + 0.1 M KIO_3 solution, respectively (The dilution of (b) solution is three times of (a) solution).

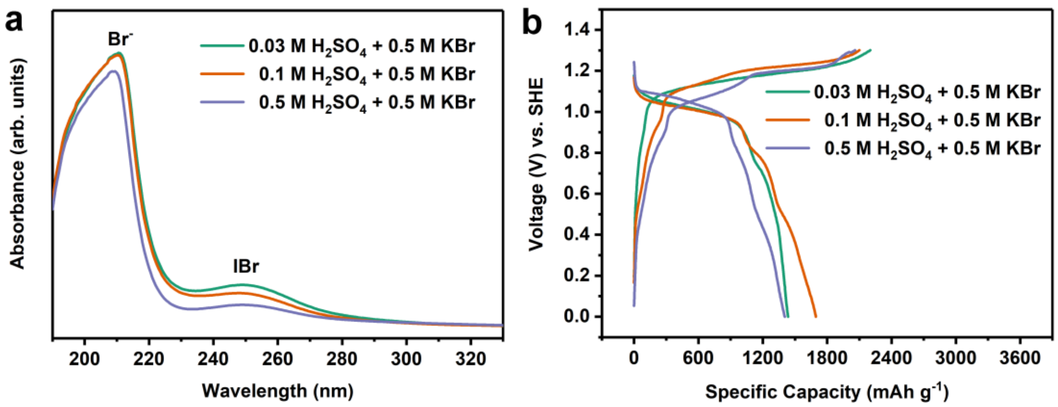

Supplementary Fig. 17 High bromide concentrations impeded the formation of IO_3^-. **(a)** UV-vis spectra and **(b)** voltage profiles of 0.5 M KBr electrolyte with different H_2SO_4 concentrations when charged to 1.3 V at 1 A g^{-1}.

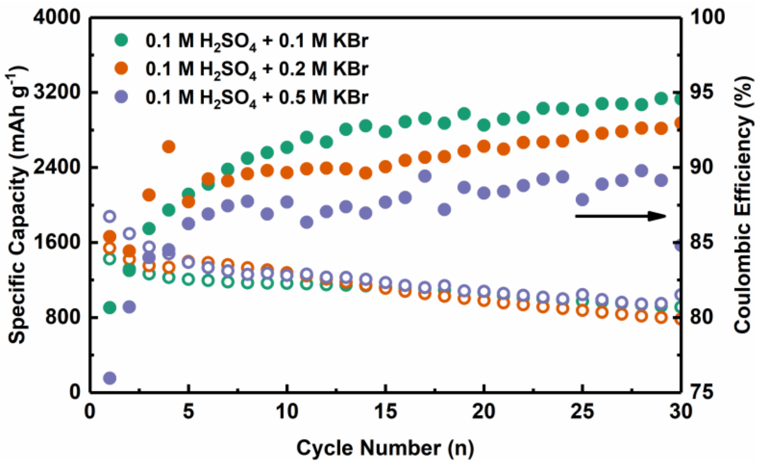

Supplementary Fig. 18 Cycling performance of I_2/HAC electrode in 0.1 M H_2SO_4 electrolyte with different KBr concentrations at 1 A g^{-1}.

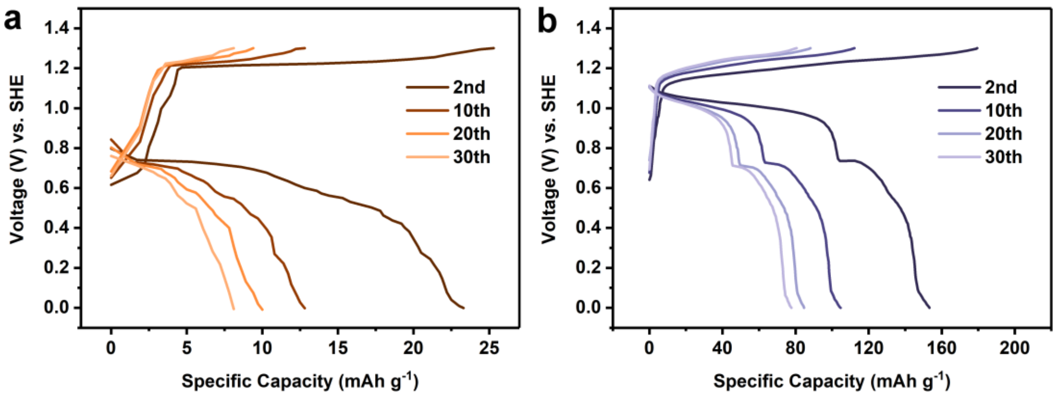

Supplementary Fig. 19 Voltage profiles of I_2/CB electrode in **(a)** 0.1 M H_2SO_4 and **(b)** 0.1 M H_2SO_4 + 0.1 M KBr electrolyte at 1 A g^{-1}.

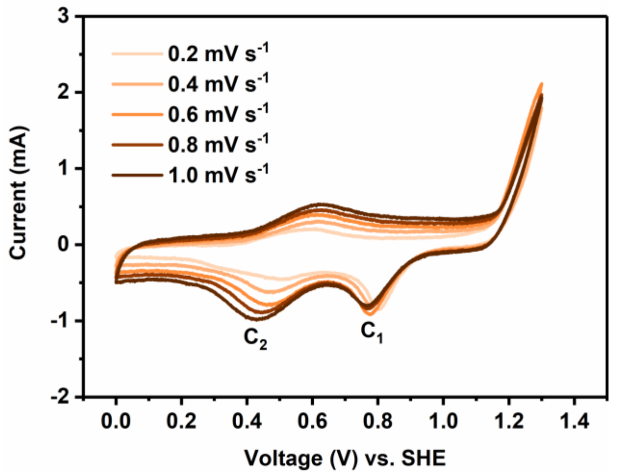

Supplementary Fig. 20 CV curves of I_2/HAC electrode in 0.1 M H_2SO_4 electrolyte at different sweep rates.

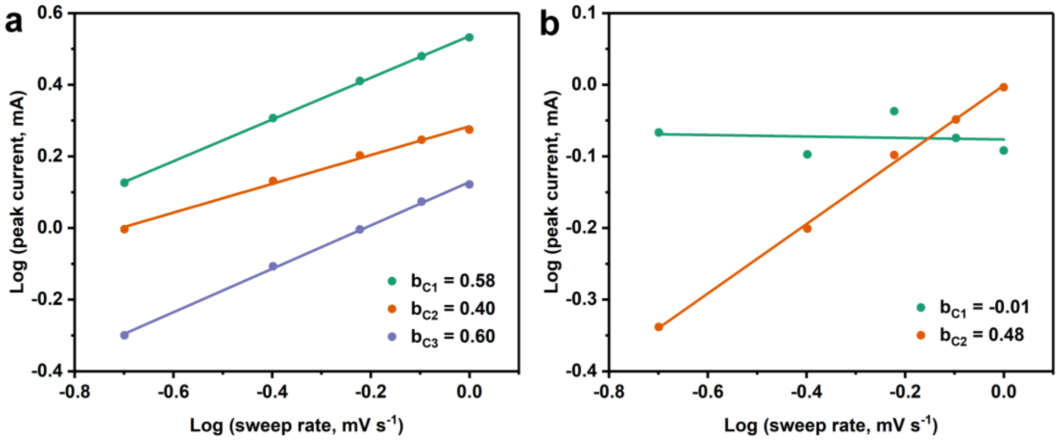

Supplementary Fig. 21 The plots of log i vs. log v, data was collected from CV curves of **(a)** 0.1 M H_2SO_4 + 0.1 M KBr and **(b)** 0.1 M H_2SO_4 electrolyte.

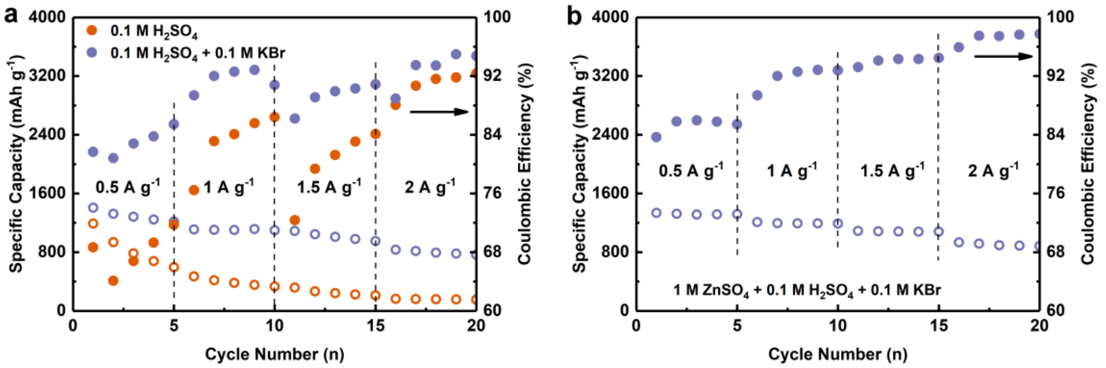

Supplementary Fig. 22 Rate performance of I_2/HAC electrode at 0.5, 1, 1.5 and 2 A g^{-1} in **(a)** 0.1 M H_2SO_4 or 0.1 M H_2SO_4 + 0.1 M KBr and **(b)** 1 M $ZnSO_4$ + 0.1 M H_2SO_4 + 0.1 M KBr electrolyte. All of the tests were collected in three-electrode cells.

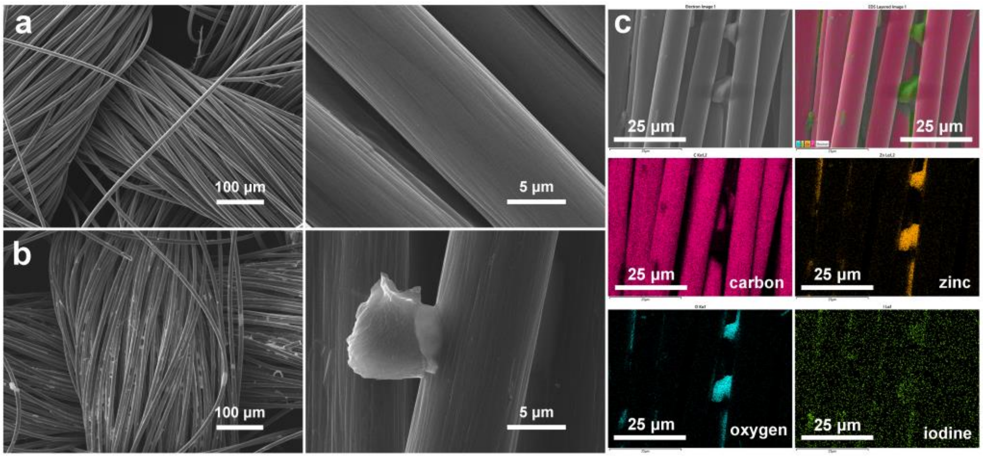

Supplementary Fig. 23 SEM images of **(a)** carbon cloth and **(b)** $Zn(IO_3)_2$ particles deposited on carbon cloth, which was collected from a full charged battery. **(c)** EDS mapping of the $Zn(IO_3)_2$ particles deposited on carbon cloth. The electrolyte is 1 M $ZnSO_4$ + 0.1 M H_2SO_4 + 0.1 M KI + 0.1 M KBr and the carbon cloth working electrode is charged to 1.3 V vs. SHE.

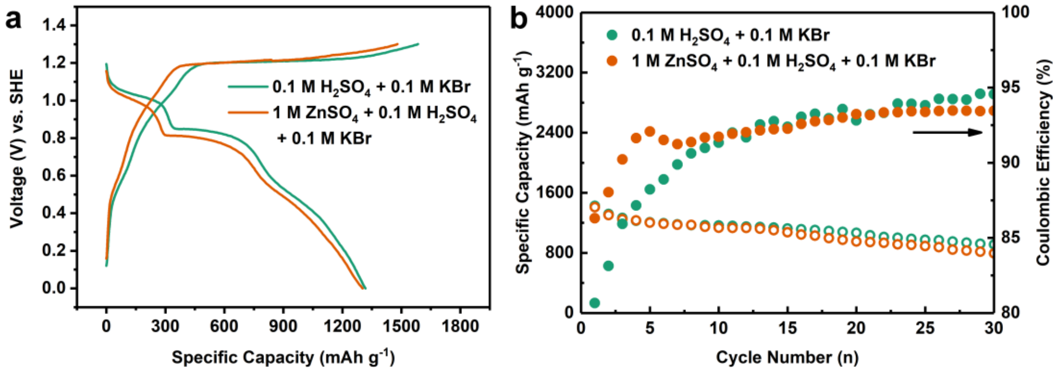

Supplementary Fig. 24 The effect of Zn^{2+} on the I^-/IO_3^- redox couple. **(a)** Voltage profiles and **(b)** cycling performance of I_2/HAC electrode at a current density of 1 A g^{-1}. The electrolyte is 0.1 M H_2SO_4 + 0.1 M KBr or 1 M $ZnSO_4$ + 0.1 M H_2SO_4 + 0.1 M KBr.

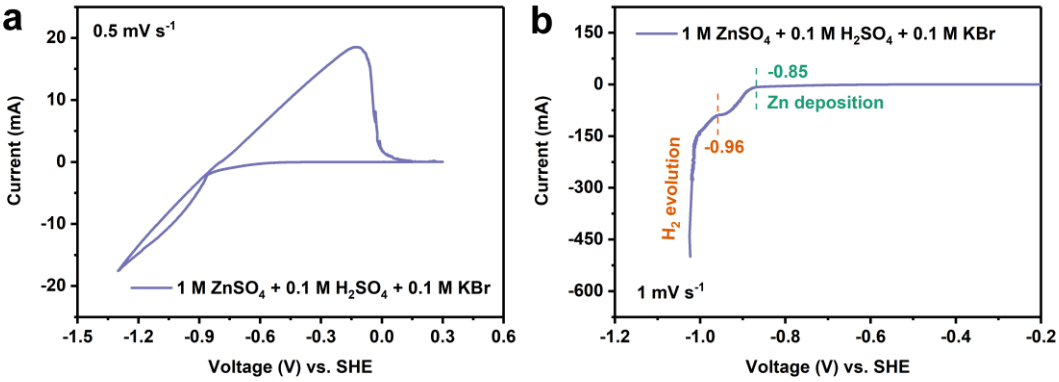

Supplementary Fig. 25 Stable zinc plating/stripping in an acidic electrolyte. **(a)** CV curve of 1 M $ZnSO_4$ + 0.1 M H_2SO_4 + 0.1 M KBr electrolyte at a sweep rate of 0.5 mV s^{-1}. The CV curve was collected in a three-electrode cell with Ti foil working electrode, Zn foil counter electrode and Hg/Hg_2SO_4 reference electrode. **(b)** LSV analysis of Zn deposition and H_2 evolution at a sweep rate of 1 mV s^{-1}.

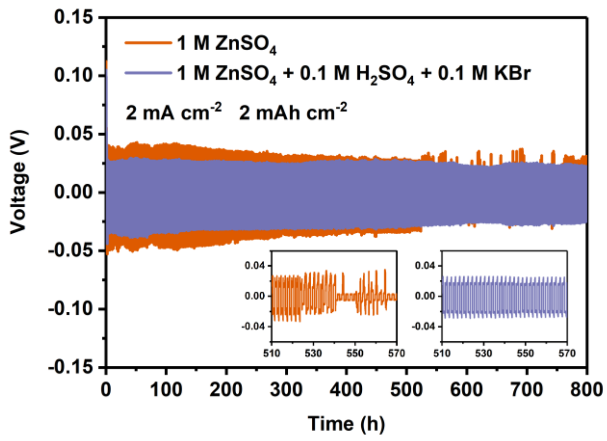

Supplementary Fig. 26 Galvanostatic Zn plating/stripping in the Zn/Zn symmetrical battery at 2 mA cm^{-2} and 2 mAh cm^{-2}.

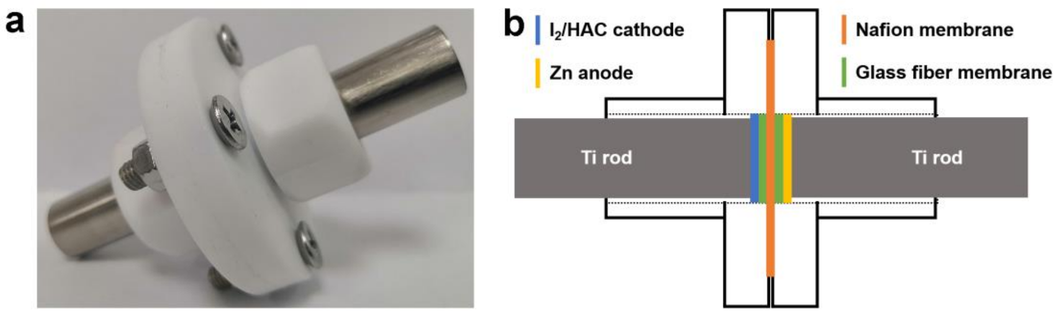

Supplementary Fig. 27 (a) The homemade two-electrode cell for Zn/I_2 full battery test. **(b)** The schematic illustration of the homemade two-electrode cell.

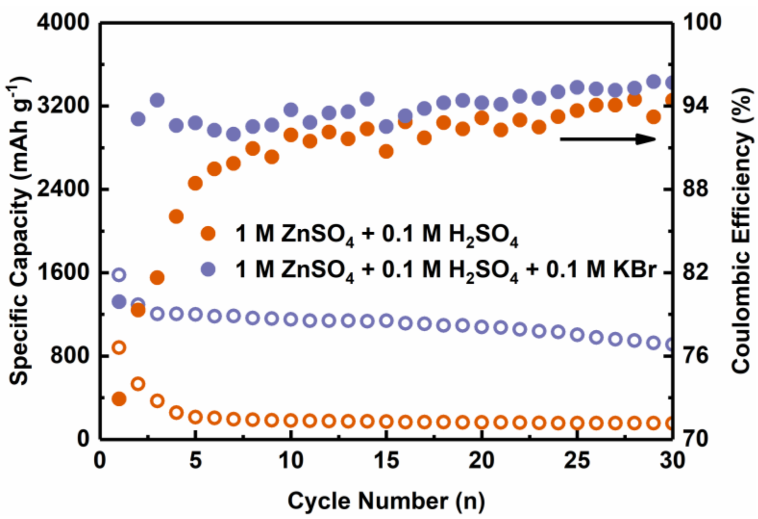

Supplementary Fig. 28 Cycling performance of Zn/I_2 full battery at 1 A g^{-1}.

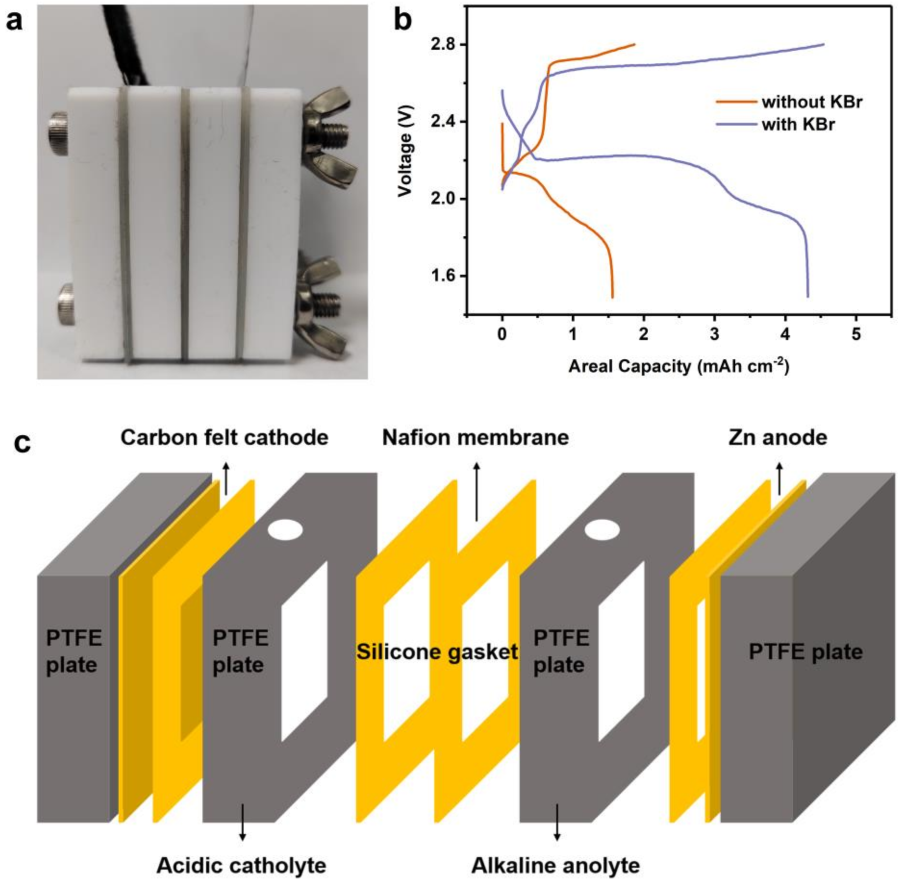

Supplementary Fig. 29 (a) An acid-alkali decoupling battery with a Zn anode, a Nafion membrane and a carbon felt cathode. The acidic electrolyte on the carbon felt cathode side is 0.1 M H_2SO_4 + 0.1 M KI or 0.1 M H_2SO_4 + 0.1 M KI + 0.1 M KBr, and the alkaline electrolyte on the Zn anode side is 6 M KOH + 0.2 M $Zn(OAc)_2$. **(b)** Voltage profiles of Zn/I_2 battery with an acid-alkali decoupling electrolyte. The current density is 3 mA cm^{-2}. **(c)** The schematic illustration of the acid-alkali decoupling battery.

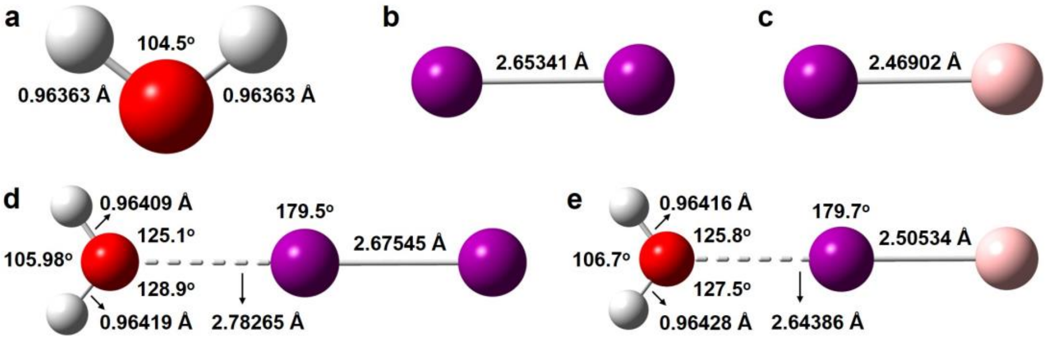

Supplementary Fig. 30 The models of the optimized structures. The bond lengths and bond angles of **(a)** H_2O, **(b)** I_2 and **(c)** IBr molecules. **(d)** The bond lengths and bond angles of H_2O and I_2 molecules when they bond to each other. **(e)** The bond lengths and bond angles of H_2O and IBr molecules when they bond to each other (the white ball represents hydrogen atom, the red ball represents oxygen atom, the purple ball represents iodine atom, and the pink ball represents bromine atom).

Supplementary Table 1 The atomic coordinates of the optimized structures.

Molecule	Atomic Number	Atomic Type	Coordinates (Å)		
			X	Y	Z
H_2O	1	O1	-0.0000	0.1179	0.0000
	2	H2	0.7621	-0.4718	0.0000
	3	H3	-0.7621	-0.4718	0.0000
I_2	1	I1	0.0000	-0.0000	1.3267
	2	I2	0.0000	-0.0000	-1.3267
IBr	1	I1	0.0000	-0.0000	0.9820
	2	Br2	0.0000	-0.0000	-1.4870
$I_2 + H_2O$	1	I1	-0.9725	-0.0056	0.0000
	2	I2	1.7029	0.0028	-0.0000
	3	O3	-3.7551	0.0102	0.0002
	4	H4	-4.3653	-0.7363	-0.0007
	5	H5	-4.3047	0.8023	-0.0006
$IBr + H_2O$	1	I1	0.0000	0.6132	-0.0000
	2	Br2	-0.0064	-1.8921	-0.0000
	3	O3	0.0199	3.2570	0.0000
	4	H4	-0.7407	3.8498	0.0000
	5	H5	0.8058	3.8155	-0.0000
